# Supplementary material for: Development of a Synthetic Hydrogel to Foster Microvascularization of an Endometriosis Microphysiological System
Source: Adv Healthc Mater. 2026 May 6;15(23):e04936. doi: 10.1002/adhm.202504936 (PMC13280185; doi:10.1002/adhm.202504936)
Supplement: Supplementary file 1 — Supporting File 1: adhm71202‐sup‐0001‐SuppMat.pdf. [file ADHM-15-0-s001.pdf]

Supporting Information

Development of a Synthetic Hydrogel to Foster Microvascularization of an Endometriosis Microphysiological System

*Lauren Pruett[A], Laura Bahlmann[A], Ryan Ogi[A], Angela Jiao[A], Priyatanu Roy[A], Matthew Johnson[B], David Trumper[B], Linda Griffith[A,B]**

[A]Department of Biological Engineering, Massachusetts Institute of Technology, Cambridge, MA, USA
[B]Department of Mechanical Engineering, Massachusetts Institute of Technology, Cambridge, MA, USA

E-mail: griff@mit.edu

Supplementary Figures:

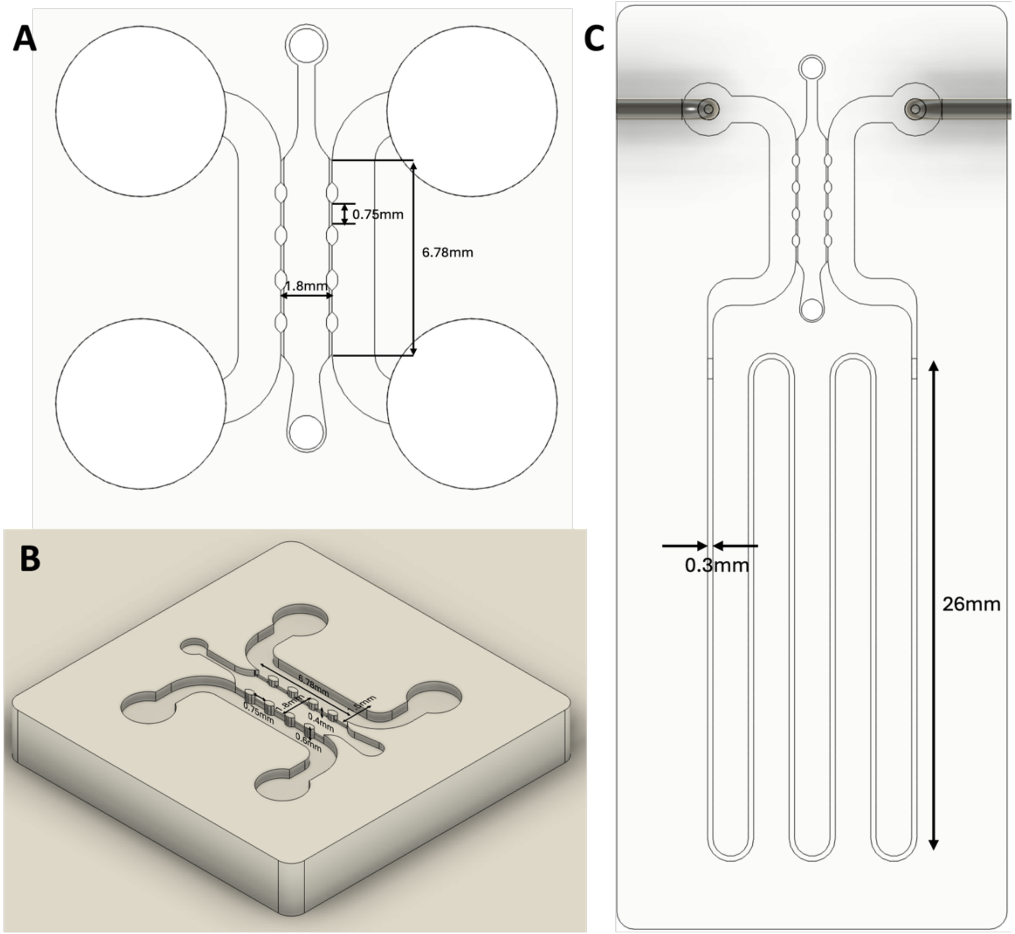

Supplemental Figure 1: CAD snapshots of custom microfluidic devices. A-B) Dimensions of the static device used in this study. C) Dimensions of the serpentine channel added to the device to enable flow studies with a continuous pressure drop. The tissue channel geometry is the same as described in A-B.

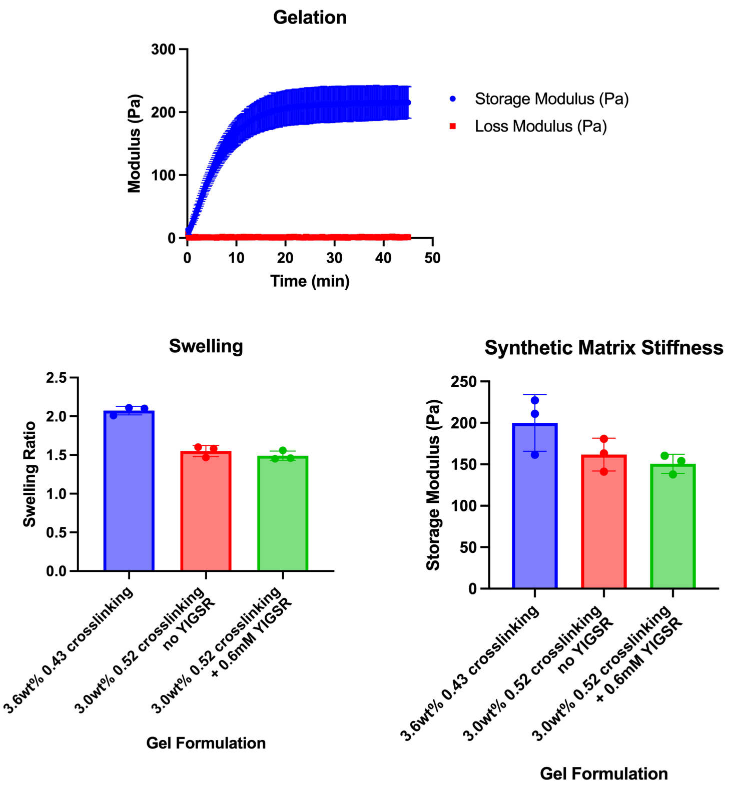

Supplemental Figure 2: Rheological and swelling characterization. A) In situ gelation curve for the 3.0wt% 0.52 crosslinking hydrogel with YIGSR shows gelation is fully complete after 30 minutes at 37°C. B) Mass swelling ratio for the three hydrogel formulations tested in this paper. The 3.0wt% 52% crosslinking hydrogel formulations swelled ~25% less compared to the original organoid formulation published in Gnecco et al[21]. C) Bulk stiffness measurements using rheology to compare the three gel formulations. The vascular formulation was ~150Pa storage modulus and 25% softer.

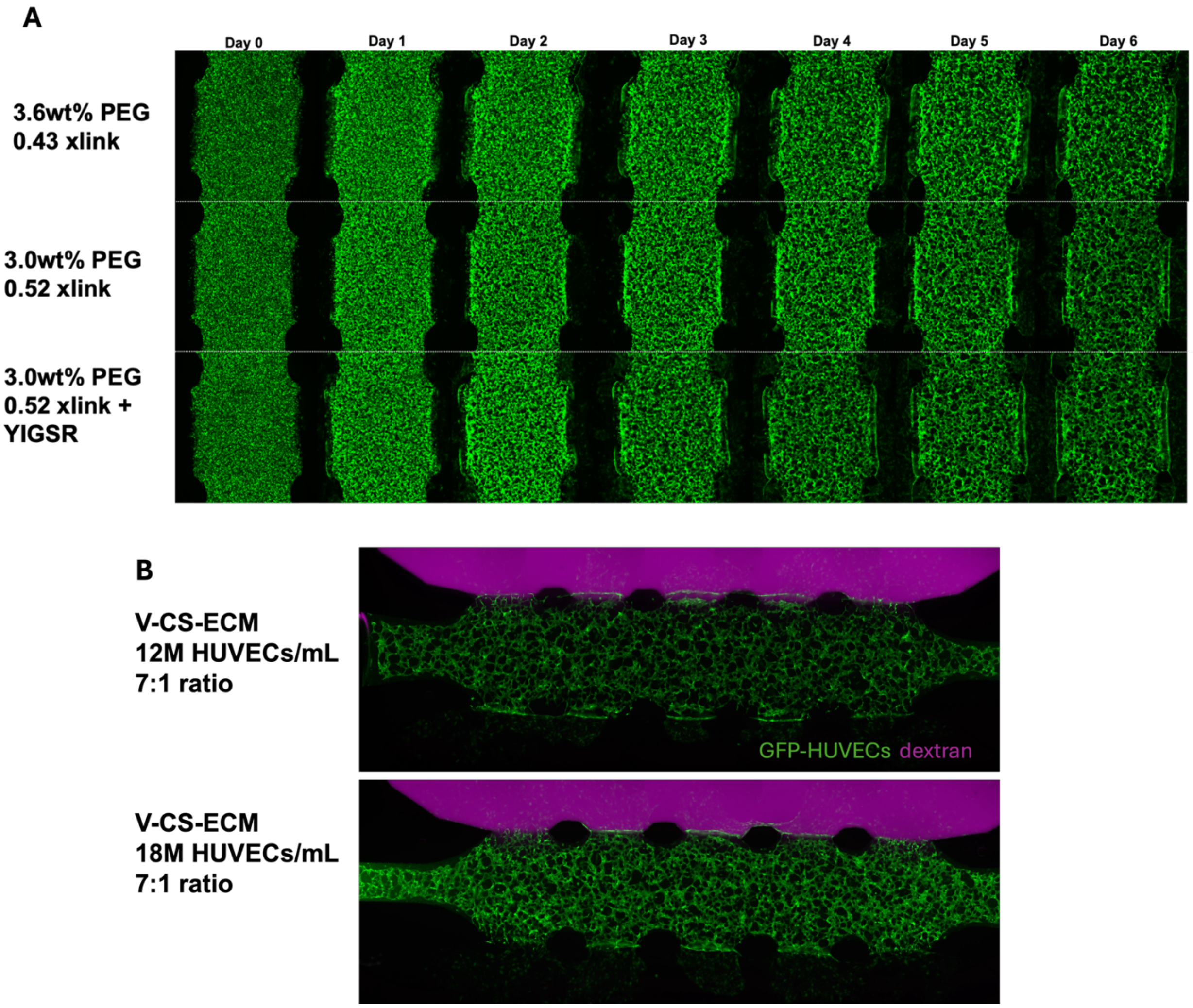

Supplemental Figure 3: Progression of network formation in static devices. A) Daily device imaging to monitor network progression in each of the different gel formulations used in this manuscript cultured in static conditions. Concentration of HUVECs: 18M/mL and 7:1 HUVEC:NHLF ratio. B) Dextran perfusion test shows that networks cultured in v-CS-ECM at both 12M HUVECs/mL and 18M HUVECs/mL did not have perfusable vessel structures.

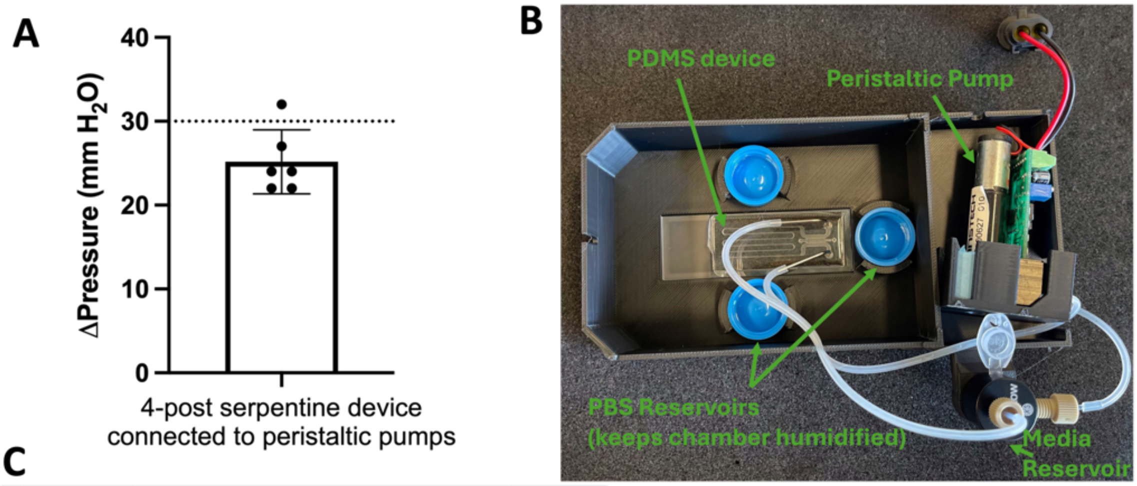

C

	Name	Components	Sterilization
1	Inlet connector	• Idex® flangeless male nut, 1/16" O.D. tubing (Cole Parmer, USA)<br>• Idex® 1/16" flangeless ferrule (Cole Parmer, USA)<br>• 70mm of Idex® high-purity PFA tubing, 0.5mm I.D. 1/16" O.D. (Cole Parmer, USA)	Ethylene Oxide
2	External tubing	• 173mm of Tygon 0.031" I.D. 0.094" O.D. (VWR, USA)	Autoclave
3	Syringe tip	• 18-gauge, 90-degree angle syringe tip (McMaster Carr, USA)	Ethylene Oxide
4	External tubing	• 165mm of Tygon 0.031" I.D. 0.094" O.D. (VWR, USA)	Autoclave
5	Barbed connectors	• Idex® 1/16" Barbed Straight Connector (Cole Parmer, USA)	Autoclave
6	Pump tubing	• 63mm of Puriflex 0.44mm I.D. autoanalysis tubing (VWR, USA)	Autoclave
7	External tubing	• 67mm of Tygon 0.031" I.D. 0.094" O.D. (VWR, USA)	Autoclave
8	Outlet connector	• Idex® flangeless male nut, 1/16" O.D. tubing (Cole Parmer, USA)<br>• Idex® 1/16" flangeless ferrule (Cole Parmer, USA)<br>• 25mm of Idex® high-purity PFA tubing, 0.5mm I.D. 1/16" O.D. (Cole Parmer, USA)	Ethylene Oxide
X	Media Reservoir	• Microfluidic Reservoir for 1.5 mL Eppendorf® - XS (Darwin Microfluidics, France)	Ethylene Oxide

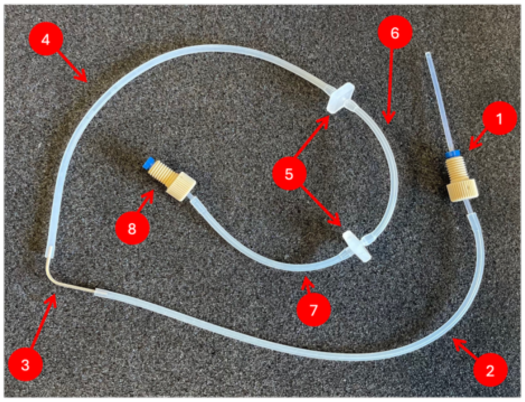

Supplemental Figure 4: Pumped device setup. A) Serpentine devices were measured to have a 25mm pressure drop across the channel. Each data point represents a different device and pump setup tested. B) Pumped experiment setup. PDMS devices were bonded to a glass slide and placed into a 3D printed holder connected to tubing and a peristaltic pump with the same footprint as a well plate to allow for easy imaging. C) List and image of components involved in the tubing setup and information for sterilization.

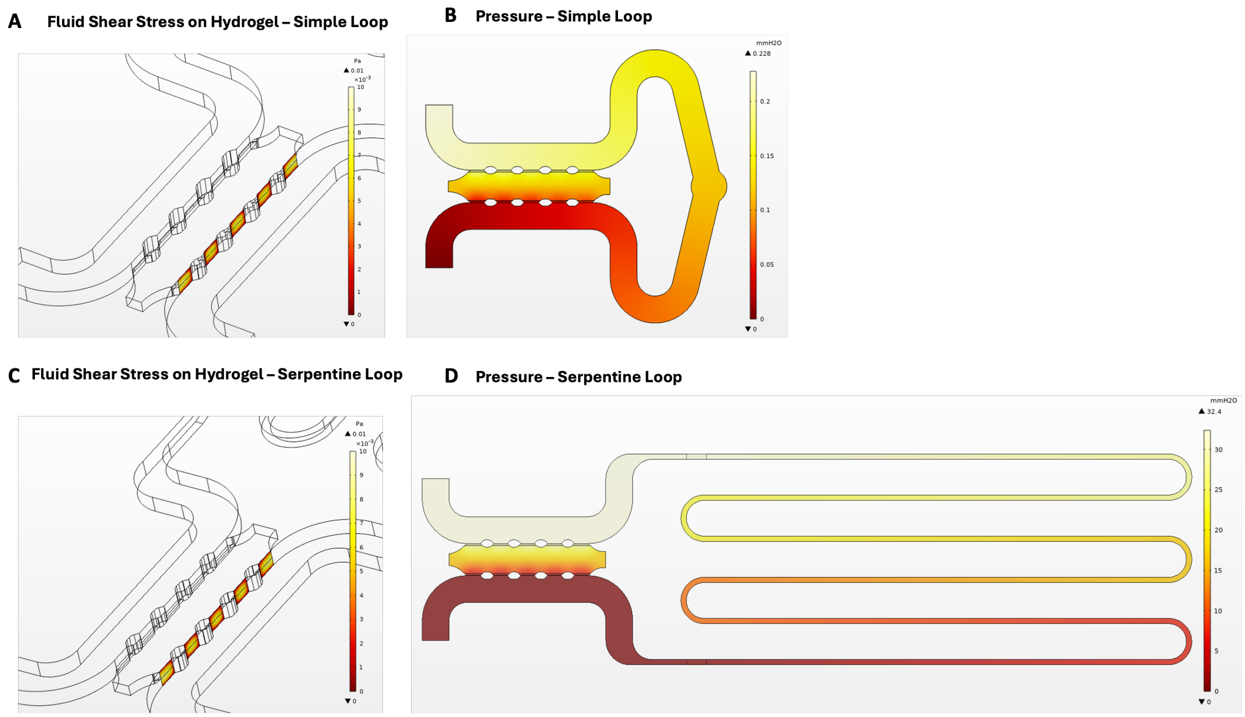

Supplemental Figure 5: Fluid shear stress and pressure drop simulations. COMSOL simulations of A) fluid shear stress on hydrogel and B) pressure in the 4-post simple-loop device used in this manuscript. COMSOL simulations of C) fluid shear stress on hydrogel and D) pressure in the 4-post serpentine-loop device used in this manuscript. Images shown are for the PEG hydrogel cases for each device and model outputs are also visualized in Table S2.

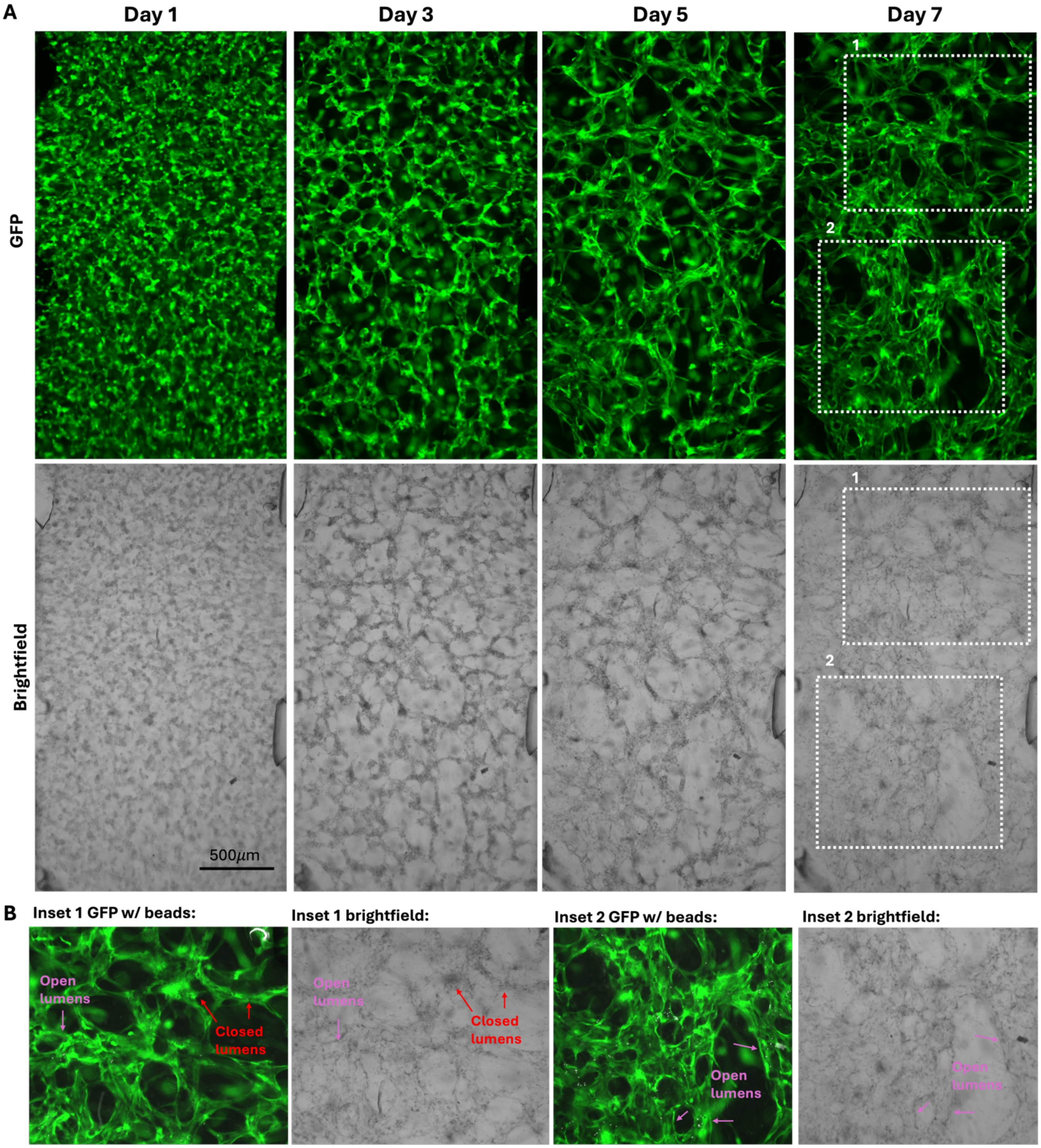

Supplemental Figure 6: Lumenization. A) Vessel formation in a representative serpentine-loop device to show emergence of open-lumen structures between day 5 and day 7, which can be visualized by perfusion with microbeads or brightfield imaging of the networks to visualize the open-lumen structures. B) Insets at day 7 demonstrating open-lumen and closed-lumen vessel structures observed in green with white microbeads (1μm) or in brightfield.

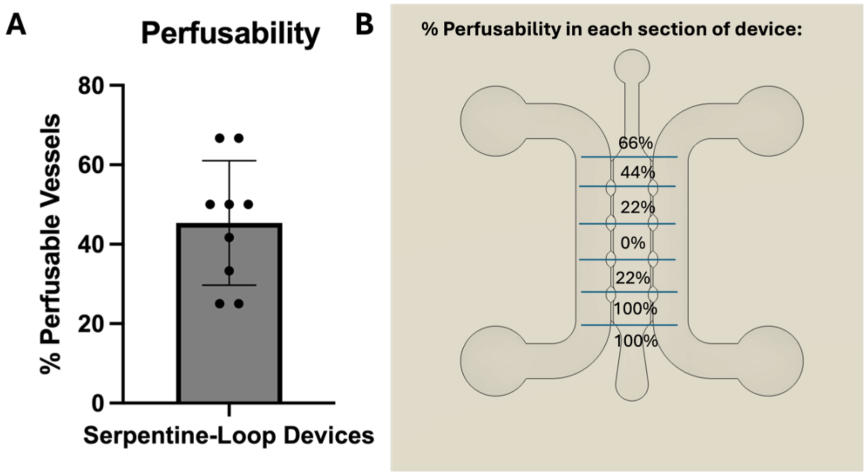

Supplemental Figure 7: Perfusability in serpentine-loop devices. A) Quantification of perfusable vessels throughout the entire device. Quantification of perfusability was performed at experiment end point between 7 and 10 days for 10 devices. B) Looking at each section of the device, we see all devices exhibiting perfusability in the bottom portion of the device, with most devices having perfusable vessels in the top and bottom of the device.

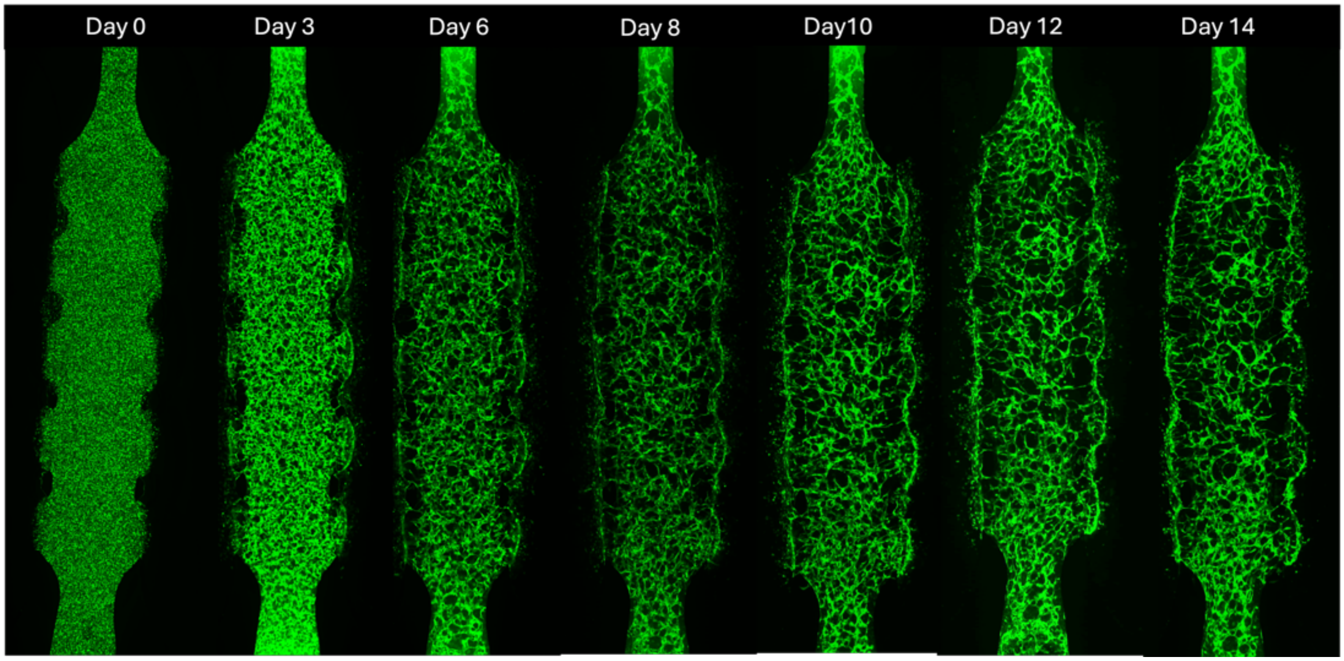

Supplemental Figure 8: Vessel stability. Vessels maintain stability for at least 14 days cultured under continuous flow.

A v-CS-ECM 15M 7:1

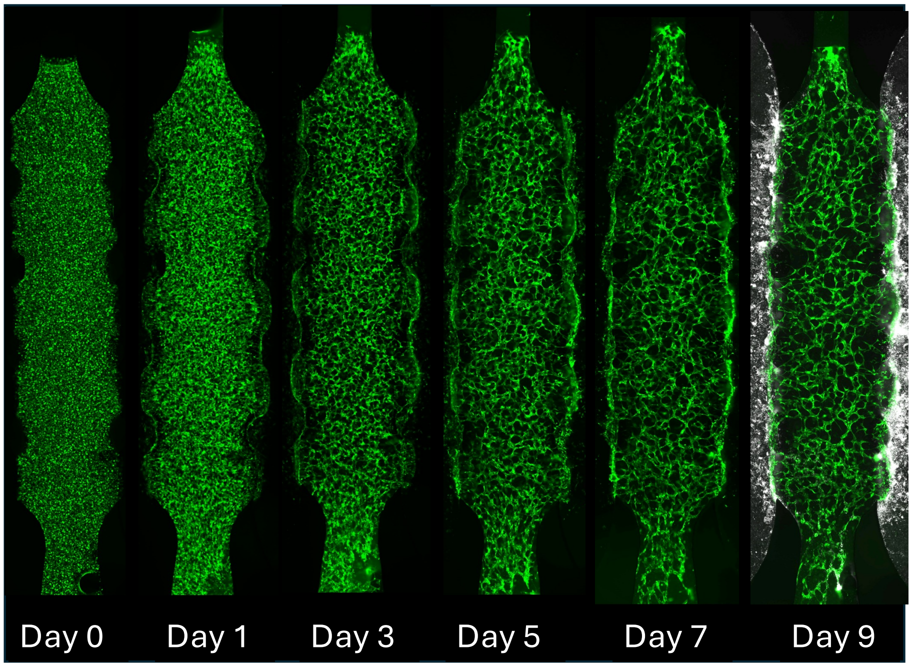

B 3.0wt% 0.52xlink no Y 18M 7:1

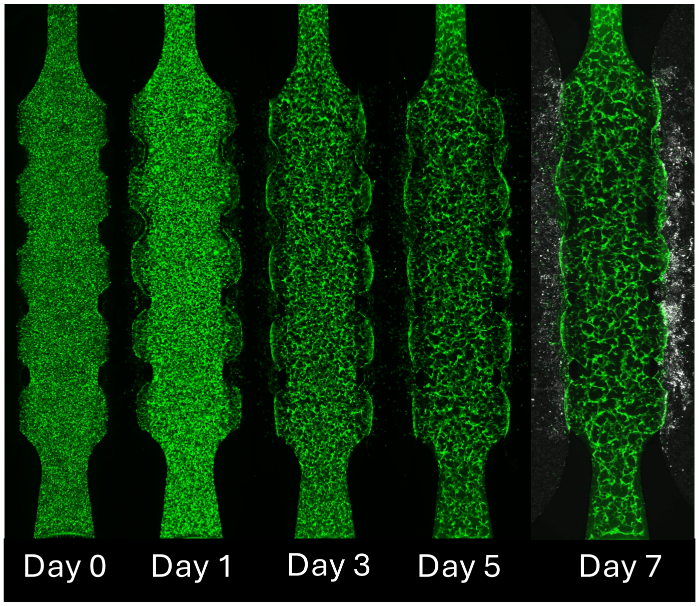

Supplemental Figure 9: Screen of formulations in serpentine-loop device. A) In attempts to decrease the concentration of endothelial cells, we tested 15M HUVECs/mL with a 7:1 HUVEC:NHLF ratio. While not as successful as 18M HUVECs/mL, perfusability was observed in the bottom quarter of the device. B) We additionally screened the softer gel formulation without YIGSR (3.0wt% 0.52xlink) with 18M HUVECs/mL and 7:1 HUVEC:NHLF ratio and observed minimal perfusability.

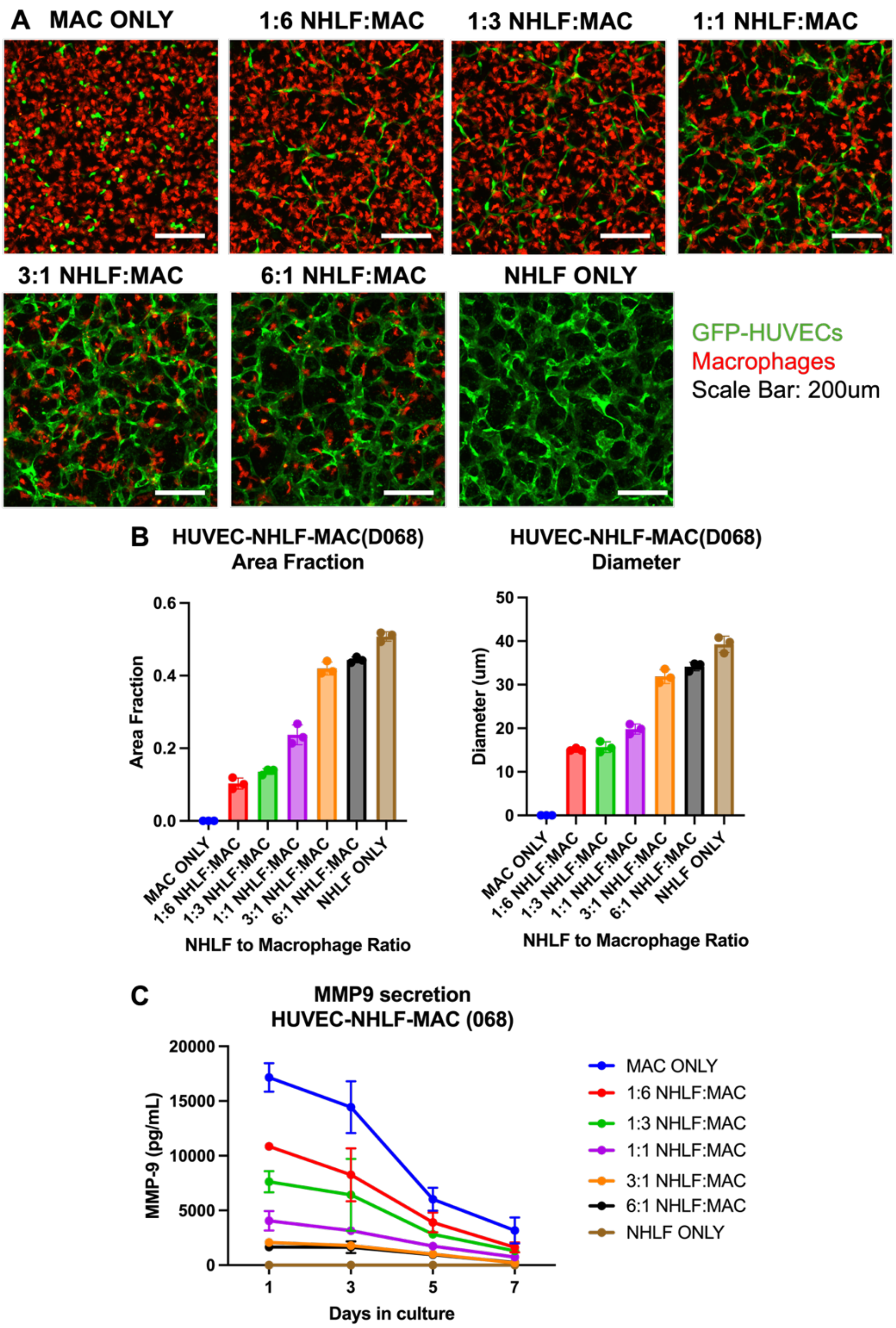

Supplemental Figure 10: Second macrophage donor in HUVEC-NHLF-macrophage networks. A) Day 5 static confocal images of HUVEC-NHLF-macrophage networks formed in static culture with varying ratios of macrophages to lung fibroblasts with a different macrophage donor. B) Increasing number of macrophages decreases area fraction and mean endothelial network segment diameter at day 5. C) Increasing MMP-9 secretion with increasing number of macrophages, demonstrating functionality. Data represented as mean±standard deviation. N=3.

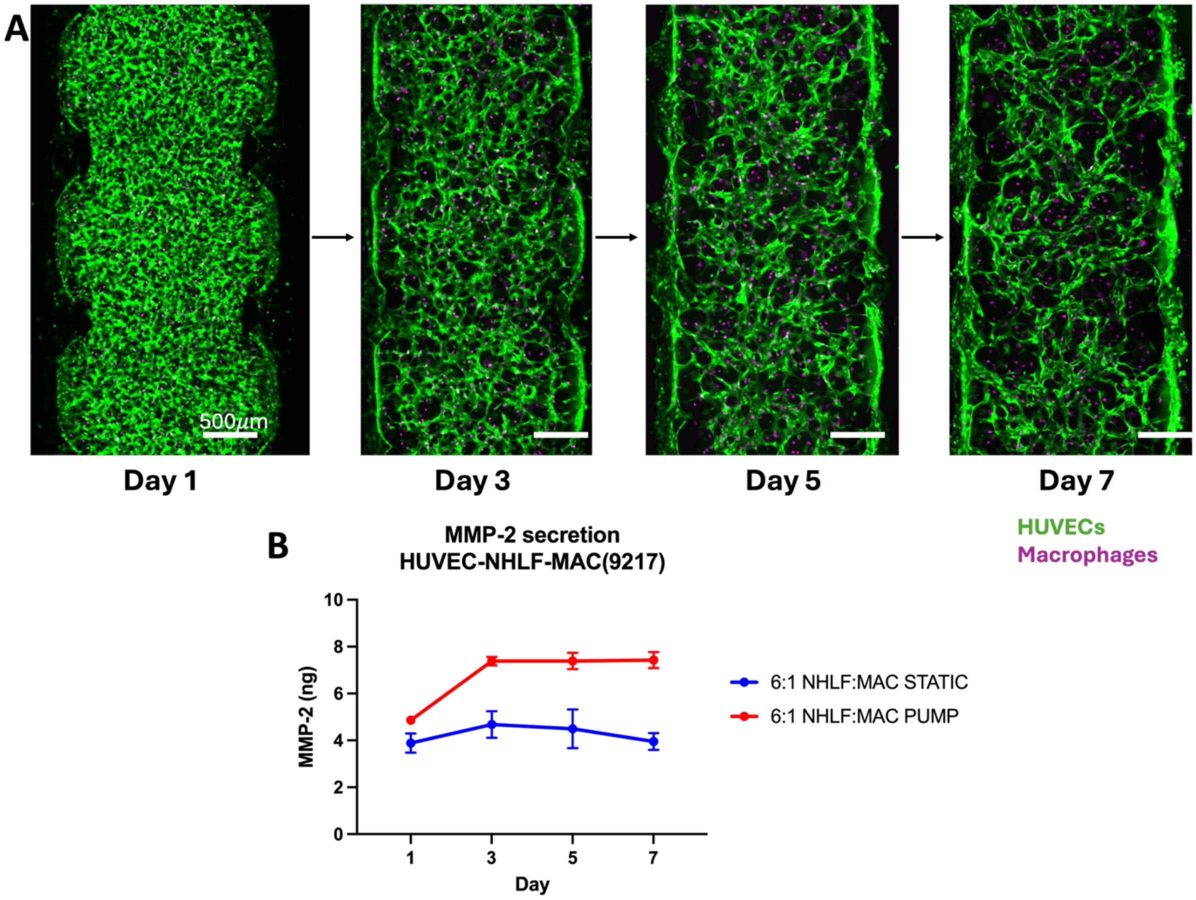

Supplemental Figure 11: Pumped HUVEC-NHLF-macrophage networks. A) Time course imaging of endothelial networks with a 6:1 nhlf to macrophage ratio in continuous pumped culture in serpentine-loop devices. B) HUVEC-NHLF-macrophage networks similarly demonstrate an increase in MMP-2 detected in the supernatant compared to static culture. Data is represented as mean ± standard deviation. N=3.

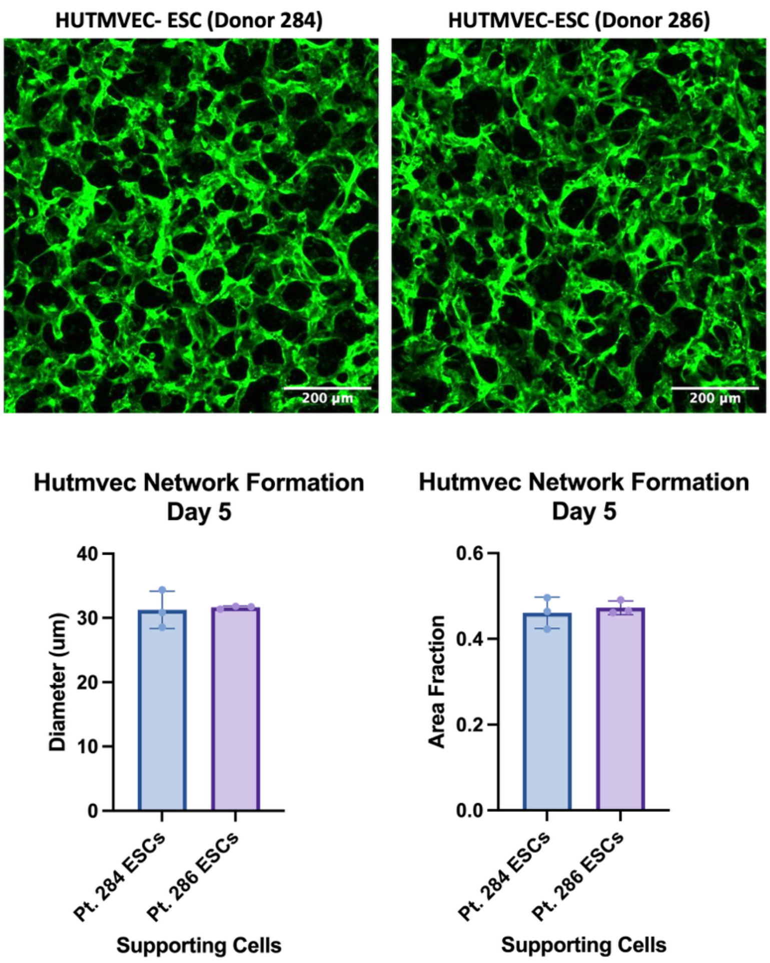

Supplemental Figure 12: HUTMVEC-ESC Network Formation with different ESC donors. HUTMVEC-ESC networks form similarly with two different endometrial stromal cell donors with no significant difference in area fraction or diameter in static culture. Data is represented as mean ± standard deviation. N=3.

A

Vasculife Basal Medium	50% base
DMEM/F12	50% base
FGF basic	2.5ng/mL
Ascorbic Acid	25 µg/mL
Hydrocortisone Hemisuccinate	0.5 µg/mL
C/S FBS	2.5%
L-Glutamine	5 mM
rhIGF-1	7.5ng/mL
rhEGF	27.5 ng/mL
rhVEGF	2.5 ng/mL
Heparin Sulfate	0.375 U/mL
Gentamicin	15 mg/mL
Amphotericin B	7.5 µg/mL
B27 Supplement	0.5x
N2 supplement	0.5x
PenStrep	0.5%
Glutamax	0.5x
ITS	0.5%
rhNoggin	50 ng/mL
Nicotinamide	0.5 mM
N-Acetyl-L-Cysteine	0.625 mM
rhFGF-10	25 ng/mL
rhRspondin-1	100 ng/mL
Estradiol	10nM

B

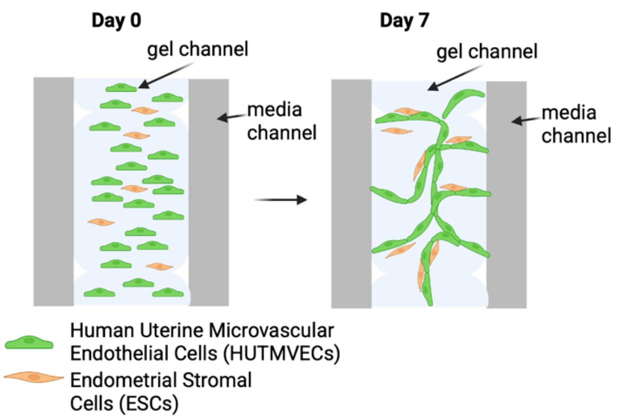

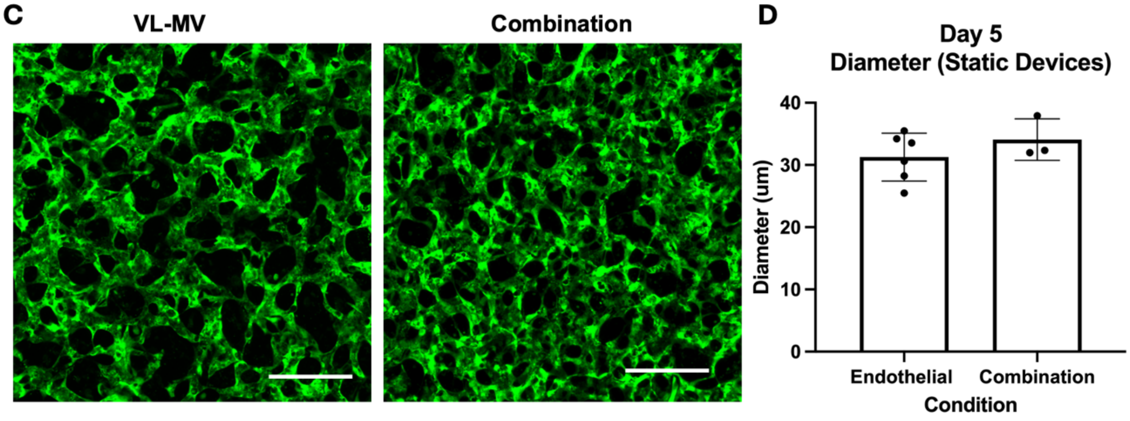

Supplemental Figure 13: Testing combination media. A) Composition of combination media. B) Using HUTMVEC-ESC culture, we tested a combination media (50% microvascular endothelial media, 50% endometrial organoid media without A8301 and Y27632) to form microvascular networks in static culture. C)Networks formed to a similar extent in the combination media D) with no significant difference in network diameters. Scale Bar: 200µm.

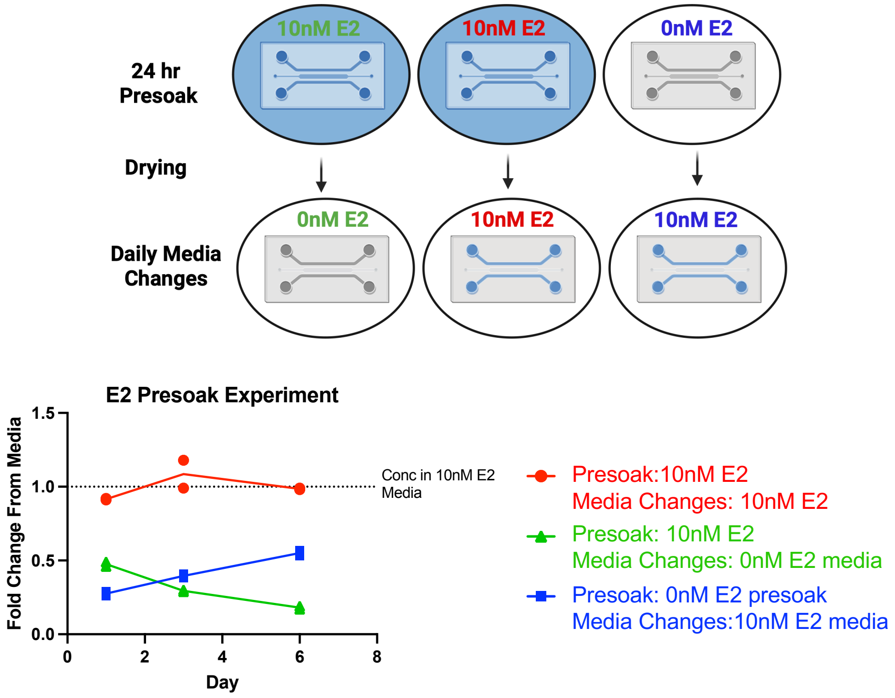

Supplemental Figure 14: E2 Presoak test. For a final experiment to combine endometrial epithelial organoids and vessels, it was necessary to use an estradiol containing medium to support organoid growth. As PDMS rapidly absorbs lipophilic compounds, it was necessary to develop a method to pre-saturate the PDMS to maintain a concentration of 10nM E2 throughout the duration of the experiment. Presoaking the devices for 24 hours in a 10nM E2 containing solution, prior to loading the gel maintained the concentration throughout the experiment by using media containing 10nM E2. If devices were not presoaked, the media only contained approximately 50% of the desired concentration by day 6. N=2 devices per condition.

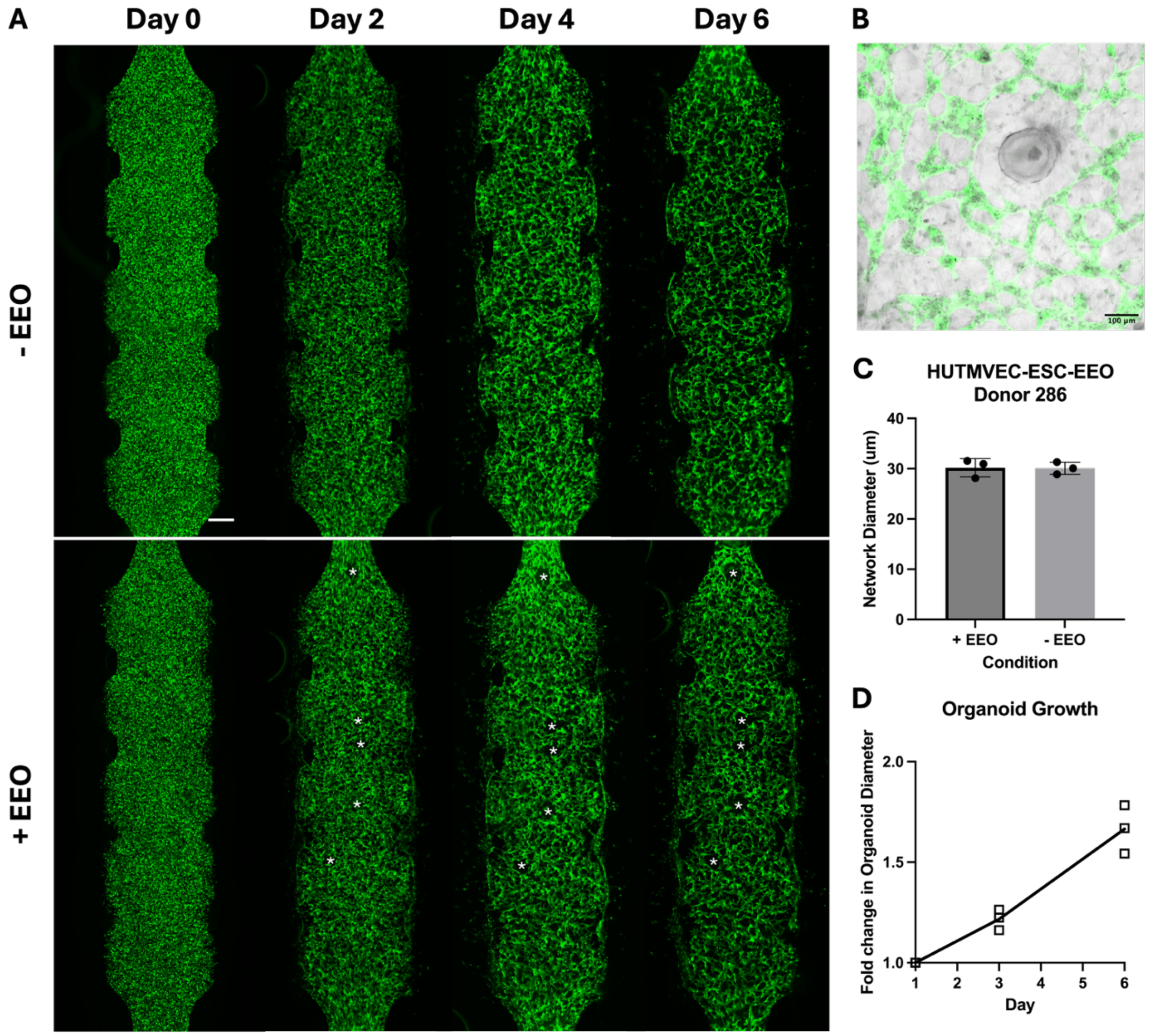

Supplemental Figure 15: EEO effects on microvessel formation. A) Network progression in serpentine-loop devices in HUTMVEC-ESC (Pt. 286) networks with and without organoids. Stars represent organoids, where it can be visualized microvessels (GFP-labeled HUTMVECs) are growing around the organoid. Scale Bar: 500μM. B) Microvessels form around organoids but are excluded from the center of the organoid. C) Diameter of the microvessels does not change compared to the no organoid condition. Images analyzed did not have an organoid in the field of view. D) Organoids grow in the microvascularized environment. N=3 devices.

Supplementary Table 1: Culture media properties and the Darcy model parameters used in the COMSOL simulation Supplemental Figure 5.

TABLE S1	Culture media viscosity	Culture media density	Hydrogel permeability
Simple loop device with fibrin	0.000862 Pa.s [1]	1002 kg/m^3 [1]	6x10^-13 m^2 [2]
Simple loop device with PEG			1x10^-17 m^2 [3]
Serpentine device with fibrin			1x10^-13 m^2 [2]
Serpentine device with PEG			1x10^-17 m^2 [3]

[1] Poon, C. Measuring the density and viscosity of culture media for optimized computational fluid dynamics analysis of *in vitro* devices. *J. Mech. Behav. Biomed. Mater.* **126**, 105024 (2022).
[2] Moreno-Arotzena, O., Meier, J. G., del Amo, C. & García-Aznar, J. M. Characterization of Fibrin and Collagen Gels for Engineering Wound Healing Models. *Materials* **8**, 1636–1651 (2015).
[3] Offeddu, G. S., Axpe, E., Harley, B. A. C. & Oyen, M. L. Relationship between permeability and diffusivity in polyethylene glycol hydrogels. *AIP Adv.* **8**, 105006 (2018).

Supplementary Table 2: Results of COMSOL simulations for four test cases. Results for PEG gel simulations are visualized in Supplemental Figure 5.

TABLE S2	Average pressure drop across the media channel	Average shear stress on gel-media interface	Interstitial flow through the hydrogel as a fraction of total media recirculation rate
Simple loop device with fibrin	0.2 mmH$_2$O	2x10^-3 Pa	0.12%
Simple loop device with PEG	0.2 mmH$_2$O	2x10^-3 Pa	2.1x10^-6%
Serpentine device with fibrin	31.0 mmH$_2$O	4x10^-3 Pa	19%
Serpentine device with PEG	32.4 mmH$_2$O	4x10^-3 Pa	4.0x10^-4%
